# Chemical characterization, antimicrobial, antioxidant, and cytotoxic potentials of *Swietenia mahagoni*

**DOI:** 10.1186/s13568-022-01406-w

**Published:** 2022-06-15

**Authors:** Sohier M. Syame, Samy M. Mohamed, E. A. Elgabry, Yousof A. A. Darwish, Asmaa S. Mansour

**Affiliations:** 1 Department of Microbiology and Immunology, National Research Centre, Dokki, 12622 Cairo Egypt; 2 Department of Medicinal and Aromatic Plants, National Research Centre, Dokki, 12622 Cairo Egypt; 3 Faculty of Medicine, Alexandria University, Alexandria, Egypt

**Keywords:** *Swietenia mahagoni* (L.) leaves, Phytochemical screening, GC/MS analysis, Antimicrobial, Antioxidant, Cytotoxic activity

## Abstract

Infectious diseases are the most important cause of death worldwide. Many of these diseases show great resistance to drugs and antibiotics with long-term use. Extracts of some medicinal plants have antimicrobial properties, which can treat and overcome these diseases. Meliaceae is a family of timber trees used extensively in treating many bacterial and fungal diseases, especially *Swietenia mahagoni* (L.) Jacq. In this study, phytochemical screening, gas chromatography/mass spectrometry (GC/MS) analysis, and antimicrobial, antioxidant, and antitumor activities of the methanolic extract of *S. mahagoni* (L.) leaves were performed. Phytochemical screening exhibited the presence of alkaloids, flavonoids, saponins, phenols, triterpenoids, glycosides, and tannins. GC/MS analysis exhibited 40 compounds, mainly 7-hexadecene, (Z)-, imidazole-4,5-d2, and 1-acetyl-4,4-bis[4-(3-bromopropoxy)-3,5-dimethoxyphenyl] piperidine. The antibacterial and antifungal potentials of the methanolic extract of *S. mahagoni* (L.) leaves was investigated using the agar well diffusion technique. Potent antibacterial activity against *Staphylococcus aureus*, *Escherichia coli*, *Salmonella enterica*, *Enterobacter aerogenes*, and *Proteus vulgaris* and antifungal activity against *Aspergillus flavus*, *Aspergillus niger*, and *Candida albicans* were found. The minimum inhibitory concentration and minimum bactericidal and fungicidal concentrations ranged from 12.5 to 25 mg/mL. Antioxidant activity was studied using the free radical scavenging assay, and the IC_50_ value of the leaf extract was 69.9 µg/mL. Cytotoxic activity was screened using the 3-(4,5-dimethylthiazol-2-yl)-2,5-diphenyltetrazolium bromide assay, and the IC_50_ value was 44.2 µg/mL. These findings suggested the importance of this plant in treating some bacterial and fungal infections and cancer.

## Introduction

In recent years, natural products, such as medicinal plants, have been introduced to treat different pathogenic infections. These products have been used to overcome the drug resistance and harmful side effects of antibiotics. This forced researchers to find new antimicrobial agents with a long history of use in ethnomedicine. Plant extracts contain numerous phytochemical compounds and are safer and more inexpensive. The development of pharmaceutical products of medicinal plants encouraged the study of phytoconstituents responsible for their biological activities [Duraipandiyan et al. 2006; Jothy et al. 2012; Mazumder et al. 2008). The mahogany (Meliaceae) is a family of flowering plants. Most species are evergreen. This family includes about 50 genera and 550 species. It has been widely cultivated in South Asia and the Pacific (Arora and Kaur 2007). Many species of this family are used for treating many diseases in traditional medicine. *Swietenia mahagoni* (L.) is a member of the family Meliaceace. Seeds are used for treating malaria, eczema, diabetes, rheumatism, cold, anorexia, and blood pressure, whereas barks are used as astringent, antipyretic, and tonic (Naveen et al. 2014). Matsuse et al. (1998) reported that an HIV protease inhibitory substance is present in *S. mahagoni*.

The secondary metabolites of the plant are responsible for their valuable therapeutic properties (Nweze et al. 2004) that can be used for the production of natural antibiotics. Different classes of Meliaceae contain chemical compounds, such as triterpenoids (limonoids). More than 300 limonoids have been extracted until now, and they are more abundant in this family than in other families, including the genus *Swietenia* (Nakatani et al. 2001). Seven limonoids have been obtained from the methanolic extract of *S. mahagoni* seeds (Govindachari et al. 1999).

*S. mahagoni* contains different phytoconstituents consisting of phenolics (flavonoids and tannins), triterpenoids and tetranortriterpenoids, saponins, and alkaloids (Sukardiman 2020). Some authors isolated limonoids and swiemahogins A and BC from leaves and twigs of *Swietenia macrophylla* (Chen et al. 2010).

The aqueous, ethanolic, petroleum ether, and chloroform extracts of *S. macrophylla* leaves were found to have antibacterial potential against methicillin-resistant *Staphylococcus aureus*, *Bacillus subtilis, Pseudomonas aeruginosa,* and *Escherichia coli* and antifungal potential against *Candida* spp., *Trichophyton mentogrophytes*, *Aspergillus flavus*, and *Aspergillus niger* (Ayyappadhas et al. 2012). Another study investigated the antimicrobial potential of aqueous and different solvent extracts of *S. mahagoni* leaves against pathogenic microorganisms and found that the methanolic extract was most potent against *E. coli*, *B. subtilis*, *K. pneumoniae*, *Candida albicans*, and *A. niger* (Chiranjib et al. 2011). The antibacterial effect of volatile oils extracted from *S. macrophylla* seeds was confirmed against *Salmonella typhimurium* (Suliman et al. 2013). Triterpenoids (B,D-secolimonoids) from *Khaya senegalensis* and *S. mahagani* were studied for their antifungal activities (Govindachari et al. 1999).

The antioxidant activity of the methanolic and aqueous extracts of *S. mahagoni* seeds was studied using 1,1-diphenyl-2-picrylhydrazyl (DPPH) and hydroxyl radical scavenging activities, and the methanolic extract showed a powerful antioxidant activity compared to the aqueous extract (Hajra et al. 2011). Also, the antioxidant activity of the methanol extract of *S. macrophylla* bark was conducted by the DPPH technique, revealing a significant antioxidant activity (Masendra and Lukmandaru 2021). Moreover, this activity was screened for three species of genus *Swietenia* (*S. mahagoni, S. macrophylla*, *and Swietenia humilis*), and they were found to have significant antioxidant activity, especially *S. macrophylla* (Preciado et al. 2016).

The cytotoxic potential of *S. mahagoni* seeds was tested against the breast cancer T47D cell line using the brine shrimp lethality test. This study investigated a powerful cytotoxic effect for the fraction consisting of alkaloids, steroids, and triterpenoids with an IC_50_ value of 49.12 ppm (Tohir et al. 2020). In addition, the limonoid compound extracted from *S. macrophylla* showed strong cytotoxicity against the colon cancer cell line HCT-116, with an IC_50_ value of 55.87 μg/mL. Limonoid did not cause rupture of red blood cells in the mice, indicating no toxicity (Pinto et al. 2021).

This study was designed to conduct the phytochemical screening, GC/MS analysis, and antibacterial, antifungal, antioxidant, and cytotoxic activities of the methanolic extract of Egyptian *S. mahagoni* (L.) Jacq leaves.

## Materials and methods

### Plant material

Fresh leaves of the medicinal plant *S. mahagoni* (L.) Jacq were collected from Plants Island, Aswan Governorate, Egypt. The plant was identified by the herbarium of the National Research Centre. The leaves were washed with running tap water to remove any traces of soil particles and dirt, washed with distilled water, and dried for 15 days in the shade. Finally, the leaves were ground and sieved to get a fine powder.

### Preparation of the plant extract

Two hundred grams of the dried plant material were taken in a conical flask and soaked with methanol at a ratio of 1:20 for 36 h at a temperature not exceeding the boiling point of methanol. The obtained extract was filtered through Whatman No. 1 filter paper and concentrated by a rotary evaporator under a vacuum at 40 °C (Atef et al. 2019). The extract was stored in a refrigerator at 4 °C and dissolved in dimethyl sulfoxide (DMSO) before use.

### Phytochemical screening

Phytochemical tests were carried out for the plant extract according to standard methods (Mamta and Jyoti 2012; Nigussie et al. 2021; Shekar et al. 2016).

#### Test for alkaloids (Mayer’s reagent test)

Diluted hydrochloric acid was added to the extract, shaken well, and filtered. A few drops of Mayer’s reagent were added to 3 mL filtrate along the sides of the tube. The formation of a creamy precipitate indicated the presence of alkaloids.

#### Test for carbohydrates (Molisch’s test)

Two drops of alcoholic α-naphthol solution were added to 2 mL aqueous extract in a test tube, and 1-mL concentrated sulfuric acid was added carefully along the sides of the test tube. The formation of a violet ring at the junction indicated the presence of carbohydrates.

#### Test for flavonoids (alkaline reagent test)

The extract was treated with a few drops of sodium hydroxide solution in a test tube. The formation of an intense yellow color, which turned colorless on the addition of a few drops of dilute acid, indicated the presence of flavonoids.

#### Test for glycosides (Keller–Killiani test)

Glacial acetic acid (3 mL) and 1 drop of 5% ferric chloride were added to a 2 mL test solution in a test tube, and 0.5 mL concentrated sulfuric acid was added to the sides of the test tube. The formation of blue color in the acetic acid layer indicated the presence of cardiac glycosides.

#### Test for tannins and phenolic compounds (ferric chloride test)

A small amount of extract was dissolved in distilled water, and 2 mL 5% ferric chloride solution were added. The formation of blue, green, or violet color indicated the presence of phenolic compounds.

#### Test for saponins (froth test)

The extract was diluted with distilled water and shaken in a graduated cylinder for 15 min. The formation of a foam layer indicated the presence of saponins.

#### Test for proteins and amino acids (Biuret test)

The extract was treated with 1 mL of 10% sodium hydroxide solution in a test tube and heated. A drop of 0.7% copper sulfate solution was added to the previous solution. The formation of violet or pink color indicated the presence of proteins.

#### Test for triterpenoids and steroids (Salkowski’s test)

The extract was treated with chloroform and filtered. Few drops of concentrated sulfuric acid were added to the filtrate, shaken, and allowed to stand. If the lower layer became red, it indicated the presence of sterol. The presence of a golden yellow layer at the bottom indicated the presence of triterpenes.

### Preparation of different plant extract concentrations

A 1% stock solution was prepared by dissolving 100-mg methanolic extract of *S. mahagoni* (L.) leaves in 10 mL DMSO. The stock solution was diluted to 50, 25, and 15 mg/mL and stored for further antimicrobial evaluation.

### GC/MS analysis of *S. mahagoni* (L.) leaf extract

GC/MS analysis was performed using a Thermo Scientific Trace GC Ultra/ISQ Single Quadrupole MS, TG-5MS fused silica capillary column (30 m, 0.251 mm, 0.1 mm film thickness). For GC/MS detection, an electron ionization system with ionization energy of 70 eV was used, and helium gas was used as the carrier gas at a constant flow rate of 1 mL/min. The injector and MS transfer line temperature was set at 280 °C.

The oven temperature was programmed at an initial temperature of 50 °C (hold for 2 min) to 150 °C at an increasing rate of 7 °C/min to 270 °C at an increasing rate of 5 °C/min (hold for 2 min) to 310 °C as a final temperature at an increasing rate of 3.5 °C/min (hold for 10 min) (Enerijiof et al. 2021). The quantification of all identified components was investigated using a percent relative peak area. Tentative identification of the compounds was performed based on the comparison of their relative retention times (RTs) and mass spectra to those of the NIST, WILLY library data of the GC/MS system.

### Antibacterial activity

#### Bacterial strains

The following bacterial strains were used in this study. *S. aureus* (ATCC 25,923) was used as Gram-positive bacteria. *E. coli* (ATCC 35218), *Salmonella enterica* (ATCC 700931), *Enterobacter aerogenes* (ATCC 13048), and *Proteus vulgaris* (ATCC 13315) were used as Gram-negative bacteria.

### Agar well diffusion assay

The bacterial strains were subcultured onto nutrient agar (Oxoid, UK) and incubated at 37 °C for 24 h. A bacterial suspension was prepared in normal saline and adjusted to 0.5 McFarland turbidity (1.5 × 10^8^ colony-forming units/mL) spread onto the Muller-Hinton agar (Oxoid) medium surface. About 100 µL of *S. mahagoni* (L.) leaf extract were poured into the wells at 50-, 25-, and 15-mg/mL concentrations. Standard antibiotics (1 mg/mL ampicillin) were used as a positive control, and methanol was used as a negative control to determine the sensitivity of the strains. The inoculated plates were incubated at 37 °C for 24 h (Syame et al. 2020). The inhibition zone diameter was measured using a ruler to determine the antibacterial activity.

### Antifungal activity

#### Fungal strains

The antifungal activity of *S. mahagoni* (L.) leaf extract was investigated against five fungal strains: *A. flavus* (ATCC 22548)*, Aspergillus fumigatus* (NCIM 1207), *A. niger* (ATCC 16888), *C. albicans* (ATCC 10231), and *Candida glabrata* (ATCC 2001).

### Agar well diffusion method

The antifungal susceptibility test was performed on Sabouraud dextrose agar (Oxoid). The inoculum suspension (0.5 McFarland) was streaked over the medium surface using a sterile cotton swab. About 100 μL of *S. mahagoni* (L.) leaf extract were poured into wells at different concentrations of 50, 25, and 15 mg/mL (Hassan and Ullah 2019). Amphotericin B (1 mg/mL) was used as a positive control to determine the sensitivity of the tested strains, and DMSO was used as a negative control. Finally, the plates were incubated at 28 °C for 48 h (for *Candida* spp.) and 72–96 h (for *Aspergillus* spp.). The inhibition zones were measured using a ruler.

### Minimum inhibitory concentration

The minimum inhibitory concentration (MIC) value was tested for the microbes sensitive to the plant extract in the agar well diffusion assay. The broth macrodilution technique was used for the determination of MIC. The plant extract was studied at different concentrations from 100 to 3.125 mg/mL in methanol. The lowest concentration with no visible turbidity in the test tube was determined as the MIC value (Sahgal et al. 2009).

### Minimum bacterial concentration and minimum fungal concentration

Minimum bacterial concentration (MBC) and minimum fungal concentration (MFC) were determined from the MIC tubes by subculturing onto Muller–Hinton (for bacteria) and Sabouraud dextrose agar (for fungi). The lowest concentration where no visible growth was observed was defined as MBC and MFC.

### Antioxidant activity

The antioxidant activity of the plant extract was assessed by the free radical scavenging activity (Chaves et al. 2020).

### Free radical scavenging activity

The free radical scavenging activity of *S. mahagoni* (L.) leaf extract was estimated using DPPH. A 0.1-mM solution of DPPH was prepared in methanol, and 1 mL of this solution was added to a 3-mL extract solution at different concentrations from 25 to 75 µg/mL. The mixture was shaken vigorously and allowed to stand at room temperature for 30 min. The absorbance was measured at 517 nm using an Asys microplate reader. The lower absorbance of the reaction mixture indicated higher free radical scavenging activity. Butylated hydroxyanisole (BHA) was used as a positive control.

$${\text{DPPH scavenging effect }}\left( \% \right) \, = { 1}00 - [\left( {\left( {{\text{A}}_{0} - {\text{ A}}_{{1}} } \right)/{\text{A}}_{0} } \right) \, \times { 1}00],$$

where A_0_ is the absorbance of the control reaction and A_1_ is the absorbance of the plant extract.

### Cytotoxic activity

#### Human cell lines

HCT-116 (colon carcinoma) and BJ-1 (normal skin fibroblast) cell lines were used in the cytotoxic assay.

#### 3-(4,5-Dimethylthiazol-2-yl)-2,5-diphenyltetrazolium bromide assay

Cell viability was assessed by the mitochondrial-dependent reduction of yellow 3-(4,5-dimethylthiazol-2-yl)-2,5-diphenyltetrazolium bromide (MTT) to purple formazan (Mosmann 1983). The cytotoxic effect of the plant extract was done in a laminar flow cabinet biosafety class II level (Baker, SG403INT, Sanford, ME, USA). Cells were suspended in RPMI 1640 medium (for HCT-116) and DMEM medium (for BJ-1) with 1% antibiotic–antimycotic mixture (10,000-U/mL potassium penicillin, 10,000-µg/mL streptomycin sulfate, and 25-µg/mL amphotericin B) and 1% L-glutamine and supplemented with 10% heat-inactivated fetal bovine serum at 37 °C under 5% CO_2_. Cells were batch cultured for 10 days and seeded at 10^4^ cells/well in fresh complete growth medium in 96-well microtiter plastic plates at 37 °C for 24 h under 5% CO_2_ using a water-jacketed carbon dioxide incubator (TC2323; Sheldon, Cornelius, OR, USA). The medium was aspirated, and fresh medium (without serum) was added with different concentrations of the plant extract to give final concentrations of 100, 50, 25,12.5, 6.25, 3.125, 1.56, and 0.78 µg/mL. After 48 h incubation, the medium was aspirated, and 40 µL MTT salt (2.5 μg/mL) was added to each well and incubated for a further 4 h at 37 °C under 5% CO_2_. To stop the reaction and dissolve the formed crystals, 200-μL 10% sodium dodecyl sulfate in deionized water was added to each well and incubated overnight at 37 °C. Adriamycin (doxorubicin) [Mw = 579.99] was used as a positive control (100 µg/mL), which gave 100% lethality under the same conditions. Cells without plant extract were used as a negative control. The absorbance was measured using a microplate multiwell reader (model 3350; Bio-Rad Laboratories, Inc., Hercules, CA, USA) at 595 nm and a reference wavelength of 620 nm (Ogbole et al. 2017).

Statistical significance was tested between the plant extract and the negative control using an independent t-test by SPSS version 11. DMSO was the vehicle used to dissolve the plant extracts, and its final concentration on the cells was < 0.2%. The percentage of change in viability was calculated according to the equation: [(Reading of extract / Reading of negative control) − 1] × 100.

### Statistical analysis

Data were analyzed using the statistical software program SPSS (version 11).

## Results

### Phytochemical screening

Qualitative analyses of the bioactive compounds for the crude methanolic extract of *S. mahagoni* (L.) leaves revealed a wide range of phytochemical compounds, as shown in Table 1. Strong positive results were found for alkaloids, flavonoids, saponins, phenols, triterpenoids, glycosides, tannins, and carbohydrates. Moderate positive results were found for steroids, amino acids, and protein, whereas negative results were found for oils.

**Table 1:** Phytochemicals of the crude methanolic extract of *S. mahagoni* (L.) leaves

No	Phytochemicals	Presence
1	Alkaloids	+ +
2	Flavonoids	+ +
3	Saponins	+ +
4	Phenols	+ +
5	Triterpenoids	+ +
6	Steroids	+
7	Glycosides	+ +
8	Tannins	+ +
9	Oils	−
10	Carbohydrates	+ +
11	Amino acids and proteins	+

### GC/MS analysis

**Fig. 1:**
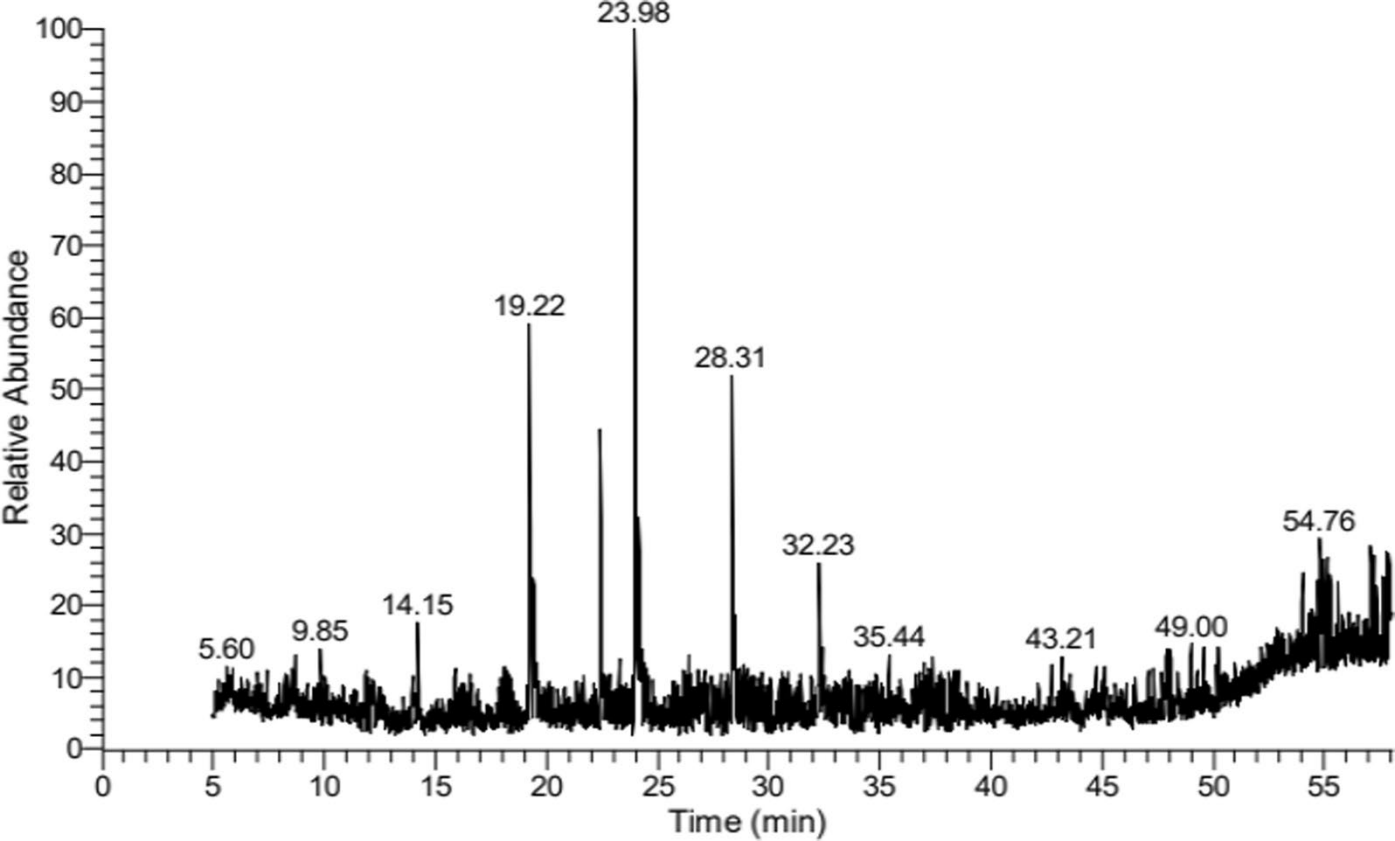
HYPERLINK "sps:id::fig1||locator::gr1||MediaObject::0" GC/MS chromatogram of the methanolic extract of *S. mahagoni* leaves

Forty compounds were characterized in the methanolic leaf extract of *S. mahagoni* (L.) by GC/MS, representing 99.99% of the extract. The main constituents are presented in Fig. 1 and Table 2, specifically 7-hexadecene, (Z)-, imidazole-4,5-d2, and 1-acetyl-4,4-bis[4-(3-bromopropoxy)-3,5-dimethoxyphenyl] piperidine with percentages of 14.41%, 10.66%, and 9.50% of the total extract, respectively.

**Table 2:** Chemical composition of the methanolic extract of *S. mahagoni* leaves

No	Compound name	RT	Peak (%)	Peak area	Molecular weight	Molecular formula
1	1-Heptanol,4-methyl-	13.99	1.69	191,833.97	130	C_8_H_18_O
2	2,4,6,8-Tetramethyl-1-undecene	14.16	2.62	297,456.95	210	C_15_H_30_
3	Imidazole-4,5-d2	19.23	10.66	1,208,060.13	68	C_3_H_2_D_2_N_2_
4	1,1,1,2-Tetrafluoro-2-tridecene	19.43	3.76	426,662.89	254	C_13_H_22_F_4_
5	Phenol,2,4-bis(1,1-dimethy)-	22.44	6.64	752,911.27	206	C_14_H_22_O
6	7-Hexadecene, (Z)-	23.99	14.41	1,632,753.67	224	C_16_H_32_
7	Oxalic acid, allyl decyl ester	24.16	4.43	501,899.75	270	C_15_H_26_O_4_
8	1-Acetyl-4,4-bis[4-(3-bromopropoxy)-3,5-dimethoxyphenyl] piperidine	28.32	9.50	1,076,909.08	671	C_29_H_39_Br_2_NO_7_
9	Dihexylsulfide	28.44	3.96	448,586.00	202	C_12_H_26_S
10	2,2′-(Buta-1,4-diyn-1,4-diyl)bis[(5,10,15,20-tetraphenylporphyrinato)zinc(II)]	30.54	2.40	271,857.54	1400	C_92_H_56_N_8_Zn_2_
11	1-Heptadecanol (CAS)	32.24	4.36	493,702.13	256	C_17_H_36_O
12	Nephthoside—1,2′,3′,4′-tetraacetate 5-(dibromomethyl)-1,3-bis(tribromomethyl) benzene	32.37	2.14	242,491.60	696	C_40_H_56_O_10_
13	(1R,2S,5R)-(1-hydroxy-2-isopropyl-5-methylcyclohexyl)acetic acid	47.83	1.56	177,060.35	214	C_12_H_22_O_3_
14	Lucenin 2	54.02	1.64	186,282.89	610	C_27_H_30_O_16_
15	1-Trimethylsilyloxy-2-trimethylsilylamino-3-(3′-methoxy-4′-trimethylsilyloxy-phenyl) propanone	54.83	1.71	193,732.19	427	C_19_H_37_NO_4_Si_3_

### Antibacterial activity

The antibacterial activity of the methanolic extract of *S. mahagoni* (L.) leaves was investigated using the agar well diffusion method, which was investigated in vitro against *S. aureus* as a Gram-positive bacterium and *E. coli*, *S. enterica*, *E. aerogenes*, and *P. vulgaris* as Gram-negative bacteria. The tested bacteria showed a variation in the inhibition patterns. The inhibition zones of the methanolic extract of *S. mahagoni* (L.) leaves at different concentrations of 50, 25, and 15 mg/mL are shown in Table 3. The extract showed a broad antibacterial spectrum against Gram- and Gram-negative bacteria at 50 mg/mL. The most susceptible microorganisms to methanolic extract were *S. aureus* (20.2 mm) and *E. aerogenes* (20.5 mm) and followed by *S. enterica* (19.5 mm), *P. vulgaris* (18.1 mm), and *E. coli* (16.4 mm). This extract showed a moderate inhibitory action at 25 mg/mL (12.2–16.3 mm) and showed a minimum inhibitory action at 15 mg/mL (5.3–8.0 mm).

**Table 3:** Antibacterial study of the methanolic extract of *S. mahagoni* (L.) leaves

Bacterial strain	50 mg/mL	25 mg/mL	15 mg/mL	Standard antibiotic (1 mg/mL ampicillin)	Methanol	MIC (mg/mL)	MBC (mg/mL)
	Inhibition zone (mm)						
*S. aureus*	20.2 ± 0.3	14.6 ± 1.2	8.0 ± 0.1	22.0 ± 0.1	NA	12.5	12.5
*E. coli*	16.4 ± 0.1	12.7 ± 0.1	5.3 ± 0.6	24.0 ± 0.3	NA	12.5	25
*S. enterica*	19.5 ± 0.4	14.0 ± 0.1	7.6 ± 1.4	24.2 ± 1.2	NA	12.5	12.5
*E. aerogenes*	20.5 ± 1.0	16.3 ± 0.5	6.0 ± 0.9	24.0 ± 0.2	NA	12.5	12.5
*P. vulgaris*	18.1 ± 0.2	12.2 ± 1.0	6.2 ± 0.3	22.3 ± 0.4	NA	12.5	25

Ampicillin: positive control, and methanol: negative control. *NA* no activity

### Antifungal activity

The antifungal activity of the methanolic extract of *S. mahagoni* (L.) leaves is illustrated in Table 4. The agar well diffusion method results indicated that *C. albicans* was the most sensitive fungal strain at the highest concentration of 50 mg/mL with the maximum inhibition zone (22.1 mm), followed by *A. flavus* (18.0 mm) and *A. niger* (17.2 mm). *A. fumigatus* and *C. glabrata* were resistant to the methanolic extract at different concentrations. At 25 mg/mL, the inhibition zone was 18.1 mm for *C. albicans*, 13.4 mm for *A. flavus*, and 12.0 mm for *A. niger*. In addition, this extract had a weak antifungal activity at 15 mg/mL with inhibition zones from 9.5 to 13.1 mm. The inhibition zones of the positive control (1 mg/mL amphotericin B) ranged from 17.2 to 24.9 mm.

**Table 4:** Antifungal study of the methanolic extract of *S. mahagoni* (L.) leaves

Fungal strain	50 mg/mL	25 mg/mL	15 mg/mL	Amphotericin B (1 mg/mL)	Methanol	MIC (mg/mL)	MFC (mg/mL)
	Inhibition zone (mm)						
*A. flavus*	18.0 ± 0.2	13.4 ± 0.3	9.5 ± 0.6	19.5 ± 2.3	NA	12.5	25
*A. niger*	17.2 ± 0.1	12.0 ± 0.5	10.0 ± 1.0	17.2 ± 0.3	NA	12.5	25
*A. fumigatus*	NA	NA	NA	20.3 ± 0.6	NA	NA	NA
*C. albicans* (ATCC 10,231)	22.1 ± 1.1	18.1 ± 0.2	13.1 ± 0.3	24.9 ± 1.1	NA	12.5	12.5
*C. glabrata*	NA	NA	NA	23.0 ± 0.2	NA	NA	NA

Amphotericin B: positive control, and methanol: negative control. *NA* no activity

### Antioxidant activity

**Fig. 2:**
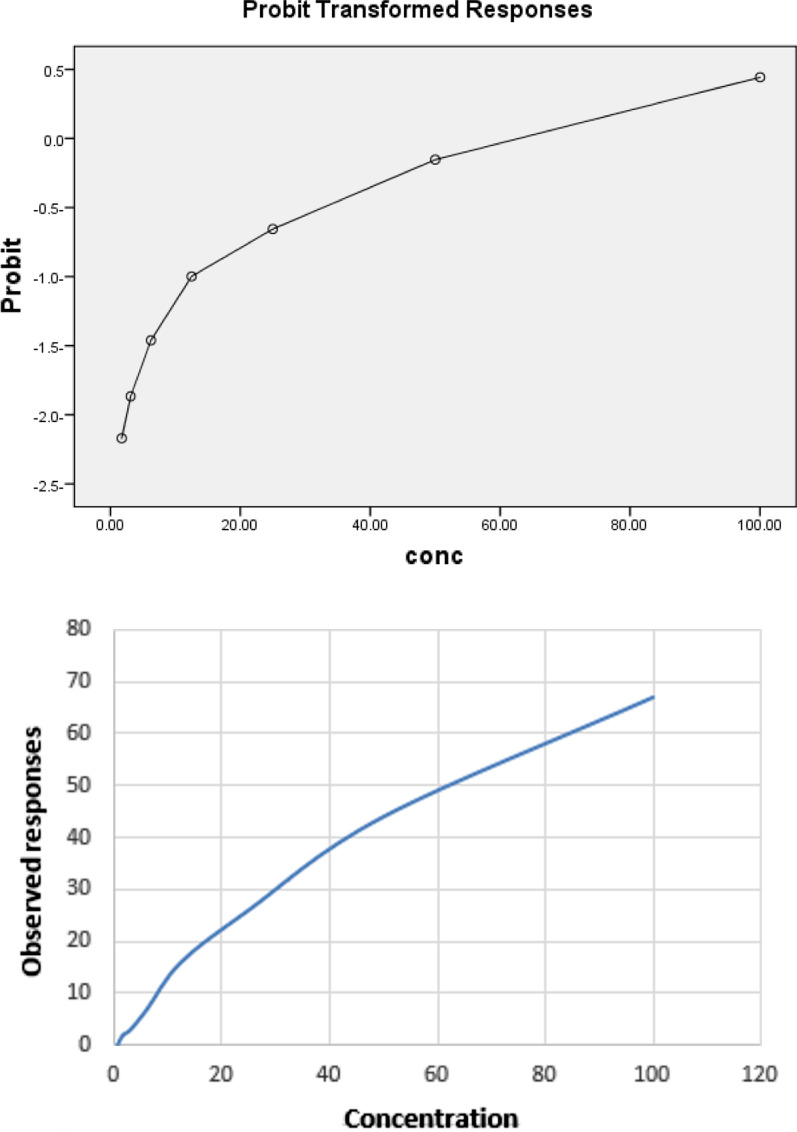
Antioxidant activity of the methanolic extract of *S. mahagoni* (L.) leaves

The free radical scavenging activity was determined by DPPH assay as illustrated in Table 5 and Fig. 2a, b. The plant extract concentrations ranged from 100 to 0.78 µg/mL. The IC_50_ value, the concentration of the plant extract causing a 50% decrease in the DPPH concentration, was calculated from the nonlinear regression curve of the log concentration of the tested plant extract (μg/mL) against the mean percentage of the radical scavenging activity. The IC_50_ value was 69.9 µg/mL for the leaf extract and 53 ± 3.1 µg/mL for the control positive (BHA). Therefore, *S. mahagoni* has a potent antioxidant activity.

**Table 5:** Antioxidant activity of the methanolic extract of *S. mahagoni* (L.) leaves

DPPH	IC_50_ (µg/mL)	Remarks
Methanolic extract of *S. mahagoni* (L.) leaves	69.9	100% at 100 µg/mL
BHA (positive control)	53 ± 3.1	–

### Cytotoxic effect

**Fig. 3:**
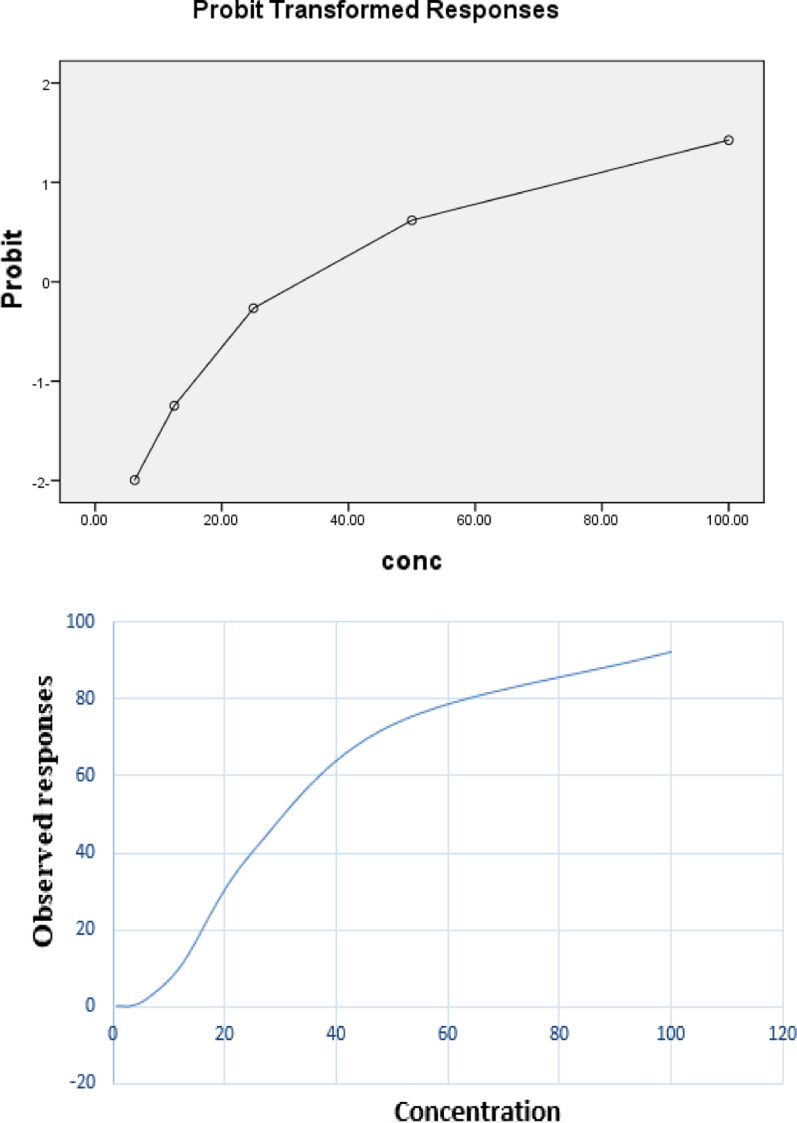
Cytotoxic effect of the methanolic extract of *S. mahagoni* (L.) leaves

The cytotoxic effect of the methanolic extract of *S. mahagoni* (L.) leaves was screened using the MTT reduction assay against the human cancer cell line HCT-116 and normal skin fibroblasts BJ-1 with different plant extract concentrations from 0.78 to 100 µg/mL, as shown in Table 6 and Fig. 3a, b. The IC_50_ values are presented in Table 6 and Fig. 2. Plant extracts showed a powerful anticancer effect against HCT-116, with an IC_50_ value of 44.2 µg/mL after incubation for 48 h. Moreover, the effect of this plant extract was tested against the normal skin fibroblast cell line and had a little effect on normal cells (20.4%) at 100 µg/mL after 48 h. Adriamycin (doxorubicin) is the drug of choice for cancer treatment, used as a positive control, and produced its anticancer effect at 37.6 µg/mL. Therefore, *S. mahagoni* leaves have a powerful anticancer compared to adriamycin.

**Table 6:** Cytotoxic effect of the methanolic extract of *S. mahagoni* (L.) leaves

Methanolic extract of *S. mahagoni* (L.) leaves	IC_50_ (µg/mL)	Remarks
HCT-116	44.2	92.3% at 100 ppm
BJ-1	–	20.4% at 100 ppm
Adriamycin (doxorubicin) [Mw = 579.99]	37.6	IC_50_ at 65.1 µM
DMSO	–	1% at 100 ppm
Negative control (cells only)	–	0%

IC_50_ = lethal sample concentration that caused the death of 50% of cells in 48 h

## Discussion

Natural products, especially medicinal plants, have been introduced in the medical field to treat different diseases due to their safety, stability, green nature, and eco-friendly effects on the environment. Moreover, these products could reduce the harmful effects caused by synthetic antimicrobials (Cantrell et al. 2012). Qualitative analyses of bioactive compounds for the methanolic extract of *S. mahagoni* leaves have been screened in this study by different standard tests, revealing a wide range of phytochemical compounds (Table 1). The most important bioactive compounds were flavonoids, alkaloids, phenolic compounds, triterpenes, and tannins [in agreement with Ekimoto et al. 1991; Nakatani et al. 2001), which have an important role in treating different infections. Other studies investigated the relationship between the phytochemical constituents of the plant extract and its biological potential (Chen et al. 2010). Some extracted some phytoconstituents (Limonoids) from the ethanolic extract of *S. mahagoni* (L.) seeds Jacq, such as 3-*O*-tigloylswietenolide and swietenine, and tested their antimicrobial activity, revealing a potent effect against *E. coli* and *Bacillus* spp. (Darussalam et al. 2014).

The antibacterial activity was studied against *S. aureus* as a Gram-positive bacterium and *E. coli*, *S. enterica*, *E. aerogenes*, and* P. vulgaris* as Gram-negative bacteria. Results found that the antibacterial activity was extremely broad against the tested bacteria at 50 mg/mL with inhibition zones from 16 to 20 mm. Besides, this plant extract showed moderate inhibition to the tested bacteria at 25 mg/mL and weak inhibition zones at 15 mg/mL. This study was supported by MIC and MBC determination, demonstrating that the plant extract showed strong activity against all tested strains, with MIC at 12.5 mg/mL and MBC at 12.5 mg/mL for *S. aureus*, *S. enterica*, and *E. aerogenes* and 25 mg/mL for *E. coli* and *P. vulgaris*. Overall, the *S. mahagoni* (L.) methanolic leaf extract possesses a broad-spectrum antibacterial activity, in agreement with other studies (Alam et al. 2014; Laxmaiah et al. 2011). The explanation of the high antibacterial activity of alcoholic extracts may be due to the high extraction efficiency of methanol with the ability to separate many active compounds responsible for antibacterial activity (Ellof 1998). These results agreed with Chiranjib et al., who found a great activity of the methanolic *S. mahagoni* leaf extract against, *E. coli*, *S. aureus, P. vulgaris*, *B. subtilis, K. pneumoniae*, *P. aeruginosa, Proteus mirabilis*, *A. fumigatus*, and *C. albicans* (Chiranjib et al. 2011)*.* Some authors also studied the methanolic leaf, stem, and root extracts against *S. aureus, B. subtilis, P. vulgaris, E. coli,* and *P. aeruginosa* and found a greater inhibitory activity (Sharma et al. 2011). Other studies reported that the antimicrobial activity of Senna, *Sesbania* flowers, and *Telosma* against *S. aureus and E. coli* might be related to the presence of phenolic compounds, including flavonoids, with percentages of 8.4%, 8.6%, and 3.4%, respectively (Krasaekoopt and Kongkarnchanatip 2005). Similar results against common foodborne pathogens were obtained in Thailand (Siriponputikorn et al. 2005). Flavonoids combined with the bacterial cell wall form a complex compound. Also, the hydroxyl groups of the phenol ring increase the hydroxylation process in bacterial cells, increasing antimicrobial effects and cytotoxicity (Cowan 1999). Another study demonstrated a biological activity for tannins that react with proline to form irretrievable compounds, preventing bacterial cell protein synthesis (Hagerman and Butler 1981).

The antifungal activity of the methanolic extract of *S. mahagoni* **(**L.) leaves was studied. *C. albicans*, *A. flavus*, and *A. niger* were the most susceptible fungal strains at 50 mg/mL with inhibition zones from 17.2 to 22.1 mm and 25 mg/mL with inhibition zones from 12.0 to 18.1 mm. Slightly less activity was detected at 15 mg/mL with inhibition zones from 9.5 to 13.1 mm. In contrast, no activity was detected against *A. fumigatus* and *C. glabrata*. In addition, the MIC and MFC were screened and found to be 12.5 and 25 mg/mL for *A. flavus* and *A. niger*, respectively. MIC and MFC of *C. albicans* were 12.5 mg/mL. These results agreed with Laxmaiah et al. (2011), who found that the methanolic extract of this plant has a strong antifungal activity against *C. albicans* and *A. niger* at 100 mg/mL with inhibition zones from 16 to 20 mm. The methanolic extract activity was tested against some fungal strains, and it had an effect against *C. albicans* and *Rhizopus* spp. (Sahgal et al. 2009). Significant antifungal activity of the *S. macrophylla* seed extract was recorded on *Fusarium* spp., *Helminthosporium* spp., and *Alternaria* spp. (Durai et al. 2016).

The antioxidant activity of the methanolic extract of *S. mahagoni* (L.) leaves was screened using the free radical scavenging assay. The leaf extract displayed a significant antioxidant potential with an IC_50_ value of 69.9 µg/mL at which 50% of the initial DPPH free radical concentration had been reduced. This extract had a potent scavenging activity in relation to the control positive (IC_50_ value of BHA = 53 ± 3.1 μg/mL). This could be owing to the high amount of phenolic, alkaloid, flavonoid, and triterpenoid compounds (Ruiz-Ruiz et al. 2017). Different solvents affected the percentage of the phytochemical compounds and therefore affected the biological activity (Ngo et al. 2017). This extract eliminated reactive oxygen species, such as peroxyl radicals, hydroxyl radicals, superoxide anions, peroxynitrite, and hypochlorous acid, and protected animal and human bodies from oxidative injuries (Chao et al. 2014). There is a relationship between antimicrobial activity and antioxidant effect, which could be due to bioactive phytochemicals (Souda et al. 2018).

The cytotoxic effect of *S. mahagoni* (L.) leaf extract was investigated against a human colon cancer cell line (HCT-116) and a normal skin fibroblast cell line (BJ-1) using the MTT assay. Live cells produce succinate dehydrogenase enzyme in the mitochondria, causing the reduction of MTT (yellow tetrazolium salt) and forming blue formazan crystals. This color was detected using spectrophotometry at a wavelength of 595 nm, so the number of live cells could be counted depending on the amount of formazan. The methanolic extract of this plant showed an IC_50_ value of 44.2 µg/mL against the HCT-116 cell line, which was slightly more than the IC_50_ value of the control positive doxorubicin (37.6 µg/mL), and it could be considered as a potential source of antitumor drugs. Moreover, this extract had very little effect on the normal skin fibroblast cell line. The anticancer effect of flavonoids (catechin and quercetin-3-O-glucoside) extracted from *S. mahagoni* (L.) leaf extract was studied against three human leukemic cell lines (U937, K562, and HL-60 cells) at very little concentrations (Roy et al. 2014). Moreover, the cytotoxic activity of the crude ethanolic extracts of *S. mahagoni* seeds, bark, and leaves was evaluated using the brine shrimp lethality bioassay, and the seed extract had the most powerful cytotoxic effect with an IC_50_ value of 4.68 µg/mL, followed by the bark extract with an IC_50_ value of 9.54 µg/mL (Akbar et al. 2009).

Overall, methanolic extract of *S. mahagoni* leaves was chemically characterized by using phytochemical screening and GC/MS analysis. This extract had a potent antibacterial activity against *S. aureus*, *E. coli*, *S. enterica*, *E. aerogenes*, and *P. vulgaris* and antifungal activity against *A. flavus*, *A. niger*, and *C. albicans.* Moreover, this extract had a significant antioxidant and antitumor activities. This is a promising natural medicinal plant for treatment of bacterial and fungal infections and cancer. Further research is required for the separation and purification of active substances for the production of efficient drugs for treating bacterial and fungal infections and cancer.

## Data Availability

All data and materials of this study are available within the article.

## Abbreviations

*S. **mahagoni*: *Swietenia* *mahagoni*
*S. aureus*: *Staphylococcus aureus*
*E. coli*: *Escherichia coli*
*S. enterica*: *Salmonella enterica*
*C. aerogenes*: *Citrobacter aerogenes*
*P. vulgaris*: *Proteus vulgaris*
*A. flavus*: *Aspergillus flavus*
*A. fumigatus*: *Aspergillus fumigatus*
*A. **niger*: *Aspergillus* *niger*
*C. albicans*: *Candida albicans*
*C. glabrata*: *Candida glabrata*
DMSO: Dimethyl sulfoxide
MIC: Minimum inhibitory concentration
MBC: Minimum bactericidal concentration
MFC: Minimum fungicidal concentration
GC/MS: Gas chromatography mass spectrometry
*S. macrophylla*: *Swietenia macrophylla*
NA: No activity
